# Performance of MALDI–TOF Mass Spectrometry in the Philippines

**DOI:** 10.3390/tropicalmed6030112

**Published:** 2021-06-26

**Authors:** Morichika Osa, Maria Cecilia Belo, Zita Dela Merced, Annavi Marie G. Villanueva, Jaira Mauhay, Alyannah Celis, Melissa Catli, Shuichi Suzuki, Tatsuya Ukawa, Shingo Tamaki, Bhim Gopal Dhoubhadel, Koya Ariyoshi, Elizabeth Freda O. Telan, Dorcas Valencia Umipig, Christopher M. Parry, Nobu Saito, Chris Smith

**Affiliations:** 1 School of Tropical Medicine and Global Health, Nagasaki University, 1-12-4, Sakamoto, Nagasaki 852-8102, Japan; nagumomoritika@yahoo.co.jp (M.O.); agvillanueva@up.edu.ph (A.M.G.V.); suzuki_shuichi@nagasaki-u.ac.jp (S.S.); t_rrss_thx@yahoo.co.jp (T.U.); b-gopal@nagasaki-u.ac.jp (B.G.D.); christopher.parry@lstmed.ac.uk (C.M.P.); nobio88@gmail.com (N.S.); 2 San Lazaro Hospital, Quiricada St., Sta. Cruz, Manila 1003, Metro Manila, Philippines; ces_belo@yahoo.com (M.C.B.); zits_dlm@yahoo.com (Z.D.M.); betelan@yahoo.com (E.F.O.T.); dorcasmd60@gmail.com (D.V.U.); 3 San Lazaro Hospital-Nagasaki University Collaborative Research Office, San Lazaro Hospital, Quiricada St., Sta. Cruz, Manila 1003, Philippines; jaira.mauhay@gmail.com (J.M.); anyacloud23@gmail.com (A.C.); ysacatli1121@gmail.com (M.C.); 4 Institute of Tropical Medicine, Nagasaki University, 1-12-4, Sakamoto, Nagasaki 852-8523, Nagasaki, Japan; smakion@me.com (S.T.); koya.ariyoshi@gmail.com (K.A.); 5 Clinical Sciences, Liverpool School of Tropical Medicine, Liverpool L3 5QA, UK; 6 Department of Microbiology, Faculty of Medicine, Oita University, Yufu, Oita 870-1192, Japan; 7 Department of Clinical Research, London School of Hygiene and Tropical Medicine, Keppel St., Bloomsbury, London WC1E 7HT, UK

**Keywords:** MALDI–TOF MS, VITEK2, biochemical methods, bacterial infection, LMIC, Philippines

## Abstract

Identification of the causative pathogen in infectious diseases is important for surveillance and to guide treatment. In low- and middle-income countries (LMIC), conventional culture and identification methods, including biochemical methods, are reference-standard. Biochemical methods can lack sensitivity and specificity and have slow turnaround times, causing delays in definitive therapy. Matrix-assisted laser desorption/ionization time of flight mass spectrometry (MALDI–TOF MS) is a rapid and accurate diagnostic method. Most studies comparing MALDI–TOF MS and biochemical methods are from high-income countries, with few reports from LMIC with tropical climates. The aim of this study was to assess the performance of MALDI–TOF MS compared to conventional methods in the Philippines. Clinical bacterial or fungal isolates were identified by both MALDI–TOF MS and automated (VITEK2) or manual biochemical methods in the San Lazaro Hospital, Metro Manila, the Philippines. The concordance between MALDI–TOF MS and automated (VITEK2) or manual biochemical methods was analyzed at the species and genus levels. In total, 3530 bacterial or fungal isolates were analyzed. The concordance rate between MALDI–TOF MS and biochemical methods was 96.2% at the species level and 99.9% at the genus level. Twenty-three isolates could not be identified by MALDI–TOF MS. In this setting, MALDI–TOF MS was accurate compared with biochemical methods, at both the genus and the species level. Additionally, MALDI–TOF MS improved the turnaround time for results. These advantages could lead to improved infection management and infection control in low- and middle-income countries, even though the initial cost is high.

## 1. Introduction

When giving treatment for bacterial infection with antibiotics, accurate identification of the causative pathogen is essential to guide their appropriate use. There are several ways to identify causative bacteria and fungi, including biochemical methods, antigen and gene detection techniques [1]. Biochemical methods, by manual tests and/or using automated equipment such as VITEK2, have been the reference-standard for the identification of bacteria in resource-limited settings. The VITEK2 system can identify bacteria automatically by reading fluorescence, turbidity and colorimetric signals. Biochemical methods usually take at least 24–48 h, including conventional culture, to identify those bacteria or fungi and can lead to delayed treatment. Matrix-assisted laser desorption/ionization time of flight mass spectrometry (MALDI–TOF MS), developed by Koichi Tanaka in 1988 [2], has become the reference-standard in many high-income laboratories [3]. MALDI–TOF MS has a high level of accuracy and provides a rapid identification (10–15 min) of microbes compared with biochemical methods [4,5,6]. MALDI–TOF MS can differentiate with high accuracy species that are difficult to be identified by biochemical methods such as *Haemophilus*, *Aggregatibacter*, *Cardiobacterium*, *Eikenella* and *Kingella* (HACEK) groups, coagulase-negative *Staphylococci* or nutritionally variant *Streptococci* [7,8,9]. MALDI–TOF MS has also been shown to be cost-effective by reducing the length of hospital admission and costs [10,11,12]. Moreover, MALDI–TOF MS has been shown to be able to predict antimicrobial resistance in bacteria [13].

Most studies comparing MALDI–OF MS and biochemical methods are from high-income countries [4,5,6], with few reports from low–middle-income countries or countries (LMIC) with tropical climates [4,14,15]. In 2015, a MALDI–TOF MS was installed in the San Lazaro Hospital (SLH)-Nagasaki Collaborative Research Laboratory and analyzed over 13,000 bacterial and fungal isolates in 5 years. The aim of this study was to assess the performance of MALDI–TOF MS compared to conventional methods in the Philippines.

## 2. Materials and Methods

This is a retrospective study using secondary data, which were collected from microbiological specimens in the San Lazaro Hospital (SLH), Metro Manila, the Philippines between 1 January 2018 and 15 January 2020. All data were de-identified to respect patient confidentiality and assigned a new code by the laboratory staff in SLH prior to being provided to the investigators.

### 2.1. Identification of Bacteria by Conventional Biochemical Methods

Bacteria or fungi cultured from clinical samples were sub-cultured for purity where necessary and examined by Gram staining and colonial morphology. Further identification was conducted using the VITEK2 compact system (version 8.01 bioMe’rieux, Marcy l’Etoile, France). In cases where the isolates could not be identified, biochemical tests that help differentiate bacteria through the characterization of their abilities in enzyme production, carbohydrate, protein, and lipid metabolism and compound utilization were performed according to standardized microbiology protocols [16].

### 2.2. Identification of Bacteria by MALDI–TOF MS

All the isolates were identified by the MALDI Biotyper 3.1 MSP database 5627 (Bruker Daltonik GmbH, Bremen, Germany). The detected spectrum was compared with reference data and evaluated by calculating a score by a unique algorithm. If the score was 2.0 or more, it was considered highly reliable at the species level, if the score was 1.7 or more and less than 2.0, it was highly reliable at the genus level, if it was less than 1.7, it was considered less reliable, and if it could not be identified, the result was ‘No identification’ returned.

## 3. Results

**Figure 1:**
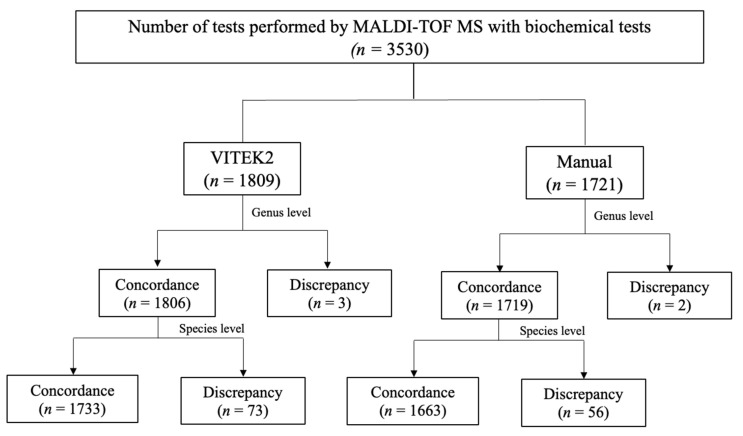
Flow chart of concordance between MALDI–TOF MS and VITEK2 or manual biochemical testing.

In total, 3530 sample isolates were tested with conventional biochemical methods. Of these, 1809 samples were tested by VITEK2, and 1721 were tested by manual methods. The concordance was calculated at the species and genus levels. Figure 1 shows the result of concordance between MALDI–TOF MS and VITEK2 or manual tests. Table 1 shows the concordance between MALDI–TOF MS and biochemical methods. The concordance was 95.8% (species level) and 99.8% (genus level) compared with VITEK2, and 96.6% (species level) and 99.9% (genus level) compared with manual biochemical testing. The total concordance was 96.2% (species) and 99.9% (genus).

The concordance of Gram-positive cocci was 100% (genus) and 97.8% (species). The concordance of Gram-negative rods was 99.8% (genus) and 95.1% (species). Among Gram-positive rods, only *Corynebacterium diphtheriae* was identified by biochemical methods. For *Corynebacterium diphtheriae*, MALDI–TOF MS had 100% concordance with the biochemical methods. Concordance of Gram-negative cocci was 100% for both genus and species. The concordance of fungi was 100% (genus) and 94.7% (species).

There were 23 isolates that could not be identified by MALDI–TOF MS. Of these, six were regarded as contaminants based on colony morphology and were not tested by biochemical methods. The remaining 17 were identified by biochemical methods: 1 was *Streptococcus pneumoniae*, three were *Pseudomonas* (1 *aeruginosa*, 1 *alcaligenes* and 1 *putida*), 1 was *Klebsiella pneumoniae*, 4 were *Cryptococcus* (3 *neoformans* and 1 *laurentii*), 7 were *Candida* (3 *albicans*, 1 *krusei*, 1 *lipolytica*, 1 *parapsilosis* and 1 *tropicalis*) and 1 was an unidentified fungus.

## 4. Discussion

This study is the first to compare the performance of MALDI–TOF MS with that of standard biochemical methods in the Philippines. MALDI–TOF MS had high concordance with biochemical methods in the identification of microorganisms at both the species and the genus levels (96.2%, 99.9%). Several studies have shown that MALDI–TOF MS is an accurate and rapid diagnostic test for not only bacteria but also fungi and acid-fast bacilli in high-income countries [17,18,19]. The finding that MALDI–TOF MS is almost equivalent to biochemical methods strongly supports its role in identifying pathogenic microorganisms in this setting. Additional methods are still sometimes needed, including biochemical methods, because MALDI–TOF MS is not good at differentiating and sometimes misidentifies closely related species [20,21,22,23]. For example, *Burkholderia pseudomallei*, *mallei*, and *thailandensis* cannot be differentiated [21], and the same is true for *Streptococcus pneumoniae* and *mitis* [16], and *Neisseria meningitidis* [23]. It may be necessary to modify the sample preparation protocol in such instances [24].

Another limitation of this method is that the accuracy of the results is highly dependent on the spectrum of the database. If an organism is not included in the database, then MALDI–TOF MS cannot identify it or sometimes misidentifies it. It is necessary that the database is up to date. Unfortunately, MALDI–TOF MS databases are proprietary, and regular database updating may not be sustainable for many laboratories, especially in LMICs. One potential solution to this is the creation of a publicly available online platform with a universal database of reference mass spectra [25]. Refining criteria, such as lowering cutoff values, for distinguishing closely related species may also be a workaround to this problem. Supplemental nucleic acid sequencing of the 16S rRNA gene may also help resolve unidentifiable or undifferentiable isolates [26]. 

In our study, 23 bacteria were not detected by MALDI–TOF MS. These results would be caused by different reasons, for example, the culture not being fresh, the colony being too small, an inadequate sample inoculated into the target plate, or the sample in the target plate being contaminated with culture media/agar.

The other disadvantage of MALDI–TOF MS is its relatively high initial cost. It may be difficult for institutions in LMIC to install this equipment, even in referral hospitals. MALDI–TOF could potentially reduce healthcare-associated costs and reduce the turnaround time for culture results, thereby allowing clinicians to initiate early targeted therapy. A previous study reported that MALDI–TOF MS is cost-effective for the identification of bacteria in an LMIC setting [14].

Even though MALDI–TOF MS displayed high accuracy for the identification of bacteria and fungi, it does not provide an answer in all circumstances. It is important to retain skills in traditional microbiological methods, and, for some microorganisms, molecular methods, such as nucleic acid sequencing, may be the best route to their identification. Furthermore, at present, MALDI–TOF MS does not provide antimicrobial sensitivity test results, and other methods to determine this property will continue to be required.

## 5. Conclusions

MALDI–TOF MS appears to be an accurate and rapid diagnostic method compared with biochemical methods at not only genus level but also species level. Additionally, with a result available in 10–15 min, MALDI–TOF MS can improve the turnaround time of results. Those advantages could lead to improved infection management and infection control in low- and middle-income countries.

## Figures and Tables

**Table 1:** Performance of the MALDI Biotyper in comparison to conventional methods.

Pathogen	No. of Tests Performed with Conventional Method *	No. of MALDI Biotyper with Score			Concordance with Conventional Method to Indicated Level (%)	
		≤1.699	1.700–1.999	≥2.000	Genus	Species
**Gram-positive cocci**	942	3	90	848	100	97.8
*Enterococcus faecalis*	23	0	1	22	100	95.7
*Enterococcus faecium*	29	0	0	29	100	100
*Staphylococcus aureus*	411	0	19	391	100	100
*Staphylococcus capitis*	3	0	0	3	100	100
*Staphylococcus cohnii*	1	0	1	0	100	100
*Staphylococcus epidermidis*	17	0	4	13	100	100
*Staphylococcus haemolyticus*	37	0	5	32	100	100
*Staphylococcus hominis*	53	0	1	52	100	98.9
*Staphylococcus kloosi*	1	1	0	0	100	100
*Staphylococcus lugdunensis*	1	0	0	1	100	100
*Staphylococcus saprophyticus*	7	0	0	6	100	100
*Staphylococcus warneri*	1	0	0	1	100	100
*Streptococcus agalactiae*	17	0	0	17	100	100
*Streptococcus anginosus*	4	0	1	3	100	100
*Streptococcus dysagalactiae*	21	0	0	21	100	95.5
*Streptococcus gallolyticus*	1	1	0	1	100	100
*Streptococcus intermedius*	1	0	0	1	100	100
*Streptococcus mitis*	144	1	18	125	100	100
*Streptococcus mitis spp*	1	0	1	0	100	100
*Streptococcus oralis*	67	0	20	47	100	100
*Streptococcus oralis spp*	1	0	1	0	100	100
*Streptococcus parasanguinis*	7	0	4	3	100	100
*Streptococcus peroris*	2	0	1	1	100	100
*Streptococcus pneumoniae*	48	0	10	38	100	62.5
*Streptococcus pyogenes*	31	0	1	30	100	100
*Streptococcus salivarius*	13	0	2	11	100	100
Gram-positive rod	122	1	9	112	100	100
*Clostridium tertium*	1	0	0	1	100	100
*Corynebacterium diphtheriae*	119	0	9	110	100	100
*Corynebacterium jeikeium*	1	0	0	1	100	100
*Rhodococcus equi*	1	1	0	0	100	100
Gram-negative cocci	34	0	0	34	100	100
*Moraxella catarrhalis*	21	0	0	21	100	100
*Moraxella equi*	1	0	0	1	100	100
*Moraxella osloensis*	1	0	0	1	100	100
*Neisseria gonorrhoeae*	2	0	0	2	100	100
*Neisseria meningitidis*	9	0	0	9	100	100
Gram-negative rod	2161	21	139	1999	99.8	95.1
*Achromobacter xylosoxidans*	18	1	3	14	94.4	77.8
*Acinetobacter baumannii*	331	1	9	321	100	99.4
*Acinetobacter baylyi*	5	0	3	2	100	20
*Acinetobacter calcoaceticus*	2	1	1	0	100	50
*Acinetobacter guillouiae*	2	0	1	1	100	100
*Acinetobacter haemolyticus*	3	0	0	3	100	100
*Acinetobacter junii*	13	0	4	9	100	69.2
*Acinetobacter nosocomialis*	22	0	1	21	100	36.4
*Acinetobacter pittii*	17	0	1	16	100	47.1
*Acinetobacter radioresistens*	1	1	0	0	100	100
*Acinetobacter ursingii*	12	0	0	12	100	83.3
*Aeromonas caviae*	3	0	0	3	100	100
*Aeromonas hydrophila*	2	0	0	2	100	100
*Aeromonas veronii*	1	0	0	1	100	0
*Burkholderia cenocepacia*	3	0	0	3	100	33.3
*Burkholderia cepacia*	9	0	1	8	100	100
*Burkholderia seminalis*	2	0	0	2	100	50
*Burkholderia thailandensis*	3	0	2	1	100	33.3
*Cedecea neteri*	1	0	0	1	100	0
*Citrobacter amalonaticus*	1	1	0	0	100	100
*Citrobacter freundii*	8	0	1	7	100	100
*Citrobacter koseri*	10	0	0	10	100	100
*Citrobacter sedlakii*	2	0	0	2	100	50
*Cronobacter sakazakii*	1	0	1	0	100	100
*Delftia acidovorans*	3	0	0	3	100	100
*Enterobacter asburiae*	21	0	3	18	100	57.1
Enterobacter cloacae **	81	1	3	76	100	97.5
*Enterobacter gergoviae*	1	0	0	1	100	100
*Enterobacter kobei*	8	1	2	5	100	62.5
*Escherichia coli*	166	0	4	162	100	100
*Haemophilus haemolyticus*	28	1	4	23	100	92.9
*Haemophilus influenzae*	123	1	3	119	100	99.2
*Haemophilus parahaemolyticus*	35	1	1	33	100	65.7
*Haemophilus parainfluenzae*	88	0	5	82	100	92
*Enterobacter aerogenes*	12	0	1	11	100	100
*Klebsiella oxytoca*	4	0	1	3	100	75
*Klebsiella pneumoniae*	526	2	48	476	100	99.6
*Leclercia adecarboxylata*	1	0	0	1	0	0
*Morganella morganii*	5	0	0	5	100	100
*Pantoea septica*	1	0	1	0	100	0
*Pasteurella multocida*	5	0	0	5	100	100
*Proteus mirabilis*	42	0	1	41	100	100
*Proteus vulgaris*	10	0	2	8	100	90
*Providencia rettgeri*	4	0	0	4	100	100
*Providencia stuartii*	4	0	1	3	100	100
*Pseudomonas aeruginosa*	400	5	13	382	100	99.3
*Pseudomonas anguilliseptica*	1	1	0	0	0	0
*Pseudomonas fluorescens*	1	0	0	1	100	100
*Pseudomonas fulva*	1	0	0	1	100	0
*Pseudomonas libanensis*	1	0	0	1	100	0
*Pseudomonas mendocina*	1	0	1	0	100	100
*Pseudomonas monteilii*	1	0	0	1	100	0
*Pseudomonas mosselii*	2	1	1	0	100	100
*Pseudomonas otitidis*	3	0	1	2	100	33.3
*Pseudomonas putida*	3	0	2	1	100	66.7
*Pseudomonas rhodesiae*	1	0	1	0	100	0
*Pseudomonas stutzeri*	9	0	0	9	100	88.9
*Ralstonia insidiosa*	1	0	0	1	100	100
*Ralstonia mannitolytica*	1	0	0	1	100	100
*Raoultella ornithinolytica*	2	1	0	1	100	50
*Serratia liquefaciens*	2	0	0	2	100	100
*Serratia marcescens*	11	0	3	8	100	100
*Serratia rubidaea*	1	0	0	1	100	100
*Serratia ureilytica*	2	0	0	2	100	0
*Shewanella algae*	1	0	0	1	100	100
*Shewanella putrefaciens*	1	0	0	1	100	100
*Stenotrophomonas maltophilia*	74	1	8	65	98.6	98.6
*Vibrio parahaemolyticus*	1	0	1	0	100	100
Fungi	270	15	87	169	100	94.7
*Candida albicans*	113	3	34	76	100	99.1
*Candida dubliniensis*	5	1	1	3	100	80
*Candida glabrata*	6	1	2	3	100	83.3
*Candida guilliermondii*	2	2	0	0	100	100
*Candida krusei*	4	1	0	3	100	100
*Candida lusitaniae*	1	0	1	0	100	100
*Candida orthopsilosis*	1	0	1	0	100	100
*Candida parapsilosis*	1	0	1	0	100	100
*Candida pararugosa*	1	0	0	1	100	100
*Candida tropicalis*	53	1	24	28	100	94.3
*Cryptococcus neoformans*	83	6	22	55	100	100
*Trichosporon inkin*	1	0	1	0	100	0
**Total**	**3530**	**40**	**325**	**3164**	**99.9**	**96.2**

Species highlighted in gray are considered clinically important microorganisms. * Includes isolates that were tested using both VITEK2 and manual biochemical methods, hence the total number may not meet. ** There was one isolate that lacked data.
